# On the Edge: Haptic Discrimination of Edge Sharpness

**DOI:** 10.1371/journal.pone.0073283

**Published:** 2013-09-04

**Authors:** Andy L. Skinner, Christopher Kent, Jonathan M. Rossiter, Christopher P. Benton, Martin G. M. Groen, Jan M. Noyes

**Affiliations:** 1 School of Experimental Psychology, University of Bristol, Bristol, United Kingdom; 2 Department of Engineering Mathematics, University of Bristol, Bristol, United Kingdom; VU University Amsterdam, Netherlands

## Abstract

The increasing ubiquity of haptic displays (e.g., smart phones and tablets) necessitates a better understanding of the perceptual capabilities of the human haptic system. Haptic displays will soon be capable of locally deforming to create simple 3D shapes. This study investigated the sensitivity of our haptic system to a fundamental component of shapes: edges. A novel set of eight high quality shape stimuli with test edges that varied in sharpness were fabricated in a 3D printer. In a two alternative, forced choice task, blindfolded participants were presented with two of these shapes side by side (one the reference, the other selected randomly from the remaining set of seven) and after actively exploring the test edge of each shape with the tip of their index finger, reported which shape had the sharper edge. We used a model selection approach to fit optimal psychometric functions to performance data, and from these obtained just noticeable differences and Weber fractions. In Experiment 1, participants performed the task with four different references. With sharpness defined as the angle at which one surface meets the horizontal plane, the four JNDs closely followed Weber’s Law, giving a Weber fraction of 0.11. Comparisons to previously reported Weber fractions from other haptic manipulations (e.g. amplitude of vibration) suggests we are sufficiently sensitive to changes in edge sharpness for this to be of potential utility in the design of future haptic displays. In Experiment 2, two groups of participants performed the task with a single reference but different exploration strategies; one was limited to a single touch, the other unconstrained and free to explore as they wished. As predicted, the JND in the free exploration condition was lower than that in the single touch condition, indicating exploration strategy affects sensitivity to edge sharpness.

## Introduction

While it has long been known that haptic feedback improves the usability of a haptic interface [1] current haptic feedback from everyday interfaces is still rather simplistic. For example, the typical use of haptic feedback in mobile telephone technology relies on global mechanical vibrotactile stimulation. However, an increasing awareness of the subtleties that can be conveyed through haptic interaction has led, for example, to patent applications for localised feedback and actuator driven dynamic displays [2], [3] and the use of electrovibrations has been shown to enhance user experiences of tactile displays [4]. The growing ubiquity of such technology means it is ever more important to understand the limits of the human perceptual system in order to design haptic displays at the appropriate level of complexity, resolution, and specificity. For example, a 6×6 tactile pin array is sufficient to display edge patterns at a resolution comparable to the human fingertip [5]. Thus, understanding the limits of the human haptic system has important engineering and design implications. The study reported here investigates human haptic perception of an object property that has yet to be the subject of much research [6], [7], namely, the sharpness of edges. Specifically, the study examines our ability to discriminate changes in edge sharpness by measuring the just noticeable difference (JND) of edge sharpness, and considers how this is affected by changes in haptic exploration strategy.

When we handle an object, many of its material properties provide enough information for it to be identified using haptic information alone; its volume, compliance, thermal characteristics, and surface texture all give clues to its identity [8]. But of all the object’s features, its three-dimensional shape provides perhaps the most diagnostic and reliable clue to its identity. An object’s shape can be described in terms of the curvature of its surfaces, its edges and the relationships between them [6], [9]. Effective identification of shape in the haptic modality requires an ability to detect and discriminate information from both the surfaces (and curves) and edges of objects [6], [10].

After vision, the haptic modality is the only other sense through which to perceive directly shape information. Therefore, to benefit individuals with visual impairments, it is important to design haptic displays which can convey shape information effectively. One mechanism commonly employed to display shape information is a two-dimensional, raised-line drawing, in which an outline of an object or scene or perhaps a more abstract image is ‘drawn’ in edges raised above the background (see [11] for a review). These raised-line drawings can either be presented passively to the skin, or in a way that enables individuals actively to explore them, usually by tracing the raised edges of the line-drawing with the fingertip [12]. Haptic ‘reading’ of these raised-line images requires the ability to detect the raised edges, and discriminate the lengths and curvature of these edges, and the angles between them [13].

So, haptic perception of real objects, haptic feedback emulating interactions with those objects, and the devices routinely used to display haptic information (raised line drawings), all rely heavily on the ability to detect edges. What do we know of the characteristics of physical edges and the sensitivity of our haptic system to these characteristics? If we define an edge as being formed by the juncture of two surfaces, a fundamental property of an edge will be the angle at which these surfaces meet (the apex in two dimensions and the vertex in three dimensions); in a three dimensional shape that property of an edge also relates to the psychological dimension of *sharpness*. It is therefore surprising that while a considerable number of studies have investigated haptic discrimination of 2D angles [13]–[16] and curved surfaces [17], until recently, very few studies have explicitly investigated discrimination of edge (vertex) sharpness [7].

Researchers have investigated our ability to discriminate the curvature of surfaces using stimuli that are both real [18]–[20] and virtual [7], [19], [21]. In the majority of these studies, the curvature of the test surfaces used was relatively large. On the one hand, for such large curvatures the notion of sharpness is inapplicable. On the other hand, a study of curvature discrimination with stimuli of appropriately small radii could inform us potentially about our discrimination of the sharpness of edges.

Park and colleagues [7] recently followed this line of logic when testing the discrimination of edge sharpness using a contact location display (CLD). The CLDs are haptic interface devices that enable the haptic exploration of virtual shapes by providing both kinesthetic and cutaneous feedback to the fingertip [19]. The cutaneous feedback, referred to as a ‘contact location cue’, is provided by an actuated roller that moves over the fingertip, in the same manner as the edge of an object might move over the fingertip. Provancher et al. [19] used virtually defined shapes with reference edges with radii (their measure of sharpness) varying from 2.5 to 20 mm, and reported JNDs that increased monotonically from 3.3 to 8.4 mm. To relate their findings to those of other studies of curvature discrimination, they expressed curvature discrimination thresholds as Weber fractions, which they calculated as the slope of the best fitting line relating JND to the reference stimulus that passes through the origin. The authors noted that there was a clear trend for Weber fractions from studies like theirs that used virtual stimuli to be higher (0.11 to 0.47 [7], [19], [21]) than studies that used real stimuli (0.08 to 0.11 [18]–[20]). This followed the pattern reported previously by Garcia-Hernandez et al. [16] who found an approximate 65% decrease in tactile discrimination thresholds for virtual stimuli compared with real stimuli for sinusoidal gratings, gap distance, and angle inclination. Park et al. [7] suggested this difference may, in the case of curvature, be attributable to limitations in the CLDs and haptic devices used to present virtual curves, and recommended discrimination of edge sharpness be explored with real stimuli. The aim of the current study is exactly this, namely, to calculate the discrimination thresholds for a set of edges.

Before describing our study, it is worth briefly reviewing the work on 2D angle discrimination, because it is the 3D equivalent of the apex (the vertex) that determines the sharpness of an edge. However, there is a clear distinction here: we are interested in the sharpness of 3D shapes and the previous studies on apex discrimination have typically used chevron shapes on the horizontal plane (with vertical information, the height and faces of the chevron, being non-diagnostic) and have allowed participants to explore the arms of the chevron shape, whereas our study excludes face exploration (the 3D equivalent of the chevron arms) and focusses on edge exploration in the vertical plane. Using stimuli consisting of raised chevrons in the horizontal plane, Wijntjes and Kappers [15] found discrimination thresholds for acute angles (20° reference) of 2.9° (chevron bisection, a Weber fraction of 0.15) and 6.0° (chevron line following, a Weber fraction of 0.30) demonstrating exploration strategy affected discriminability (discrimination thresholds for obtuse angles, 135° reference, nearly doubled). Importantly, Wijntjes and Kappers [15] found that local apex information was important for accurate discrimination, with thresholds lower for stimuli with apex information (6.0°) than without (7.5°). Our study focuses on local vertex information as this is what ‘sharpness’ refers to and excludes exploration of faces.

Voisin and colleagues [22], [23] did investigate angle discrimination on the vertical plane, but, instead of investigating the convex-angle, they investigated the concave angle, thus the angle does not directly relate to the psychological dimension of sharpness. They used angles cut into 1 cm thick (thus they refer to 2D angle, as there is limited scope to explore the horizontal plane) Plexiglas. In order to explore the concave angle it was necessary to investigate the faces of the Plexiglas shape, whereas in our experiments, to explore the sharpness of an edge, minimal face exploration was allowed. Nonetheless, it will be potentially informative to compare our results to those of Voisin and colleagues, who found a discrimination threshold of between 4.7° (a Weber fraction of 0.05, [14]) and 4° (a Weber fraction of 0.04, [22]). Alary and colleagues [23] reported slightly higher thresholds of 5.7° and 5.8° (for arm straight and arm flexed respectively) but similar thresholds for participants who were blind (4.3° and 4.9°; see also [13], [24]). Interestingly, Levy and colleagues [25] showed that exploration strategy made little difference to the threshold (around 6°) and that a static single touch was sufficient to gain maximal information. However, Levy [25] found that using a hand-held tool to explore the shape decreased discriminability (9.6°) suggesting that cutaneous information is more important than proprioceptive information for angle discrimination (in our study we focus on cutaneous perception, minimising proprioception). Thus, discrimination of a concave angle, like 2D acute angles, appears relatively good and can be quickly gained via a single static touch. We might expect edge sharpness of real objects to demonstrate a similar level of sensitivity as angle discrimination.

In our study, we make use of state-of-the-art 3D printing technology to produce a set of three-dimensional shape stimuli with edges that vary in sharpness. We reasoned that, while at some scale all edges are rounded at their apex, at the scale of our everyday industrialised environment, objects can appear to have edges that have no perceivable apex curvature and are sharp to the touch. Our sensitivity to changes in sharpness may be qualitatively different to our sensitivity to changes in curvature. To test this, the edges of our stimuli were true, sharp edges with no perceivable apex curvature.

As with many psychological phenomena, it is likely that our sensitivity to changes in edge sharpness will change over a range of sharpness. Indeed, an important characteristic of the perception of any class of stimuli is the way in which discrimination thresholds scale as the magnitude of stimulus scales, and classically, the extent to which this follows Weber’s Law. Our first experiment explored this, and measured the JND in edge sharpness using a number of reference edges (40°, 50°, 70° and 90°). We examine how the Weber fractions scale across these different conditions, and how these relate to the Weber fractions reported in studies of 2D angle and curvature discrimination.

Exploration strategy can have a significant impact on performance in haptic discrimination tasks [15], [25]–[27], and the second experiment reported here explores the effect of different exploration strategies on the JND of edge sharpness. Active exploration can provide superior haptic shape discrimination compared to passive exploration (where a stimulus is presented to the fingertip), probably by optimising the intake of relevant stimulus information [28], [29]. It is also the way we normally interact with the physical world around us. To capture this mode of tactile sensing, participants were permitted to actively explore the sharpness of edges. However, using active exploration still leaves a variety of ways in which stimuli can be explored, and these variations can again affect performance [30]. Understanding how performance changes with exploration strategy will provide important design constraints for the future engineering of haptic devices. To investigate this, two groups of participants repeated the task from Experiment 1 (with a single reference), but each group used a different exploration strategy. The first group used a ‘Single Touch’ strategy in which the participant presented the pad of their fingertip to the test edge in a single stationary touch from above. Haptic displays should be quick and easy to read, so a single touch may well be preferred to a more extended exploration; the findings from [25] suggested that such a strategy may indeed be sufficient. However, it is important to know what the maximal amount of information gain is in order to determine the trade-off between a brief ‘haptic glance’ and a more complex and extended exploration. Therefore, the second group of participants was permitted to use ‘Free Exploration’ to explore the edge using whatever strategy they wished. Subsequently, the JNDs for the two strategies were compared.

## Methods

### Ethics Statement

Both Experiments were approved by the Faculty of Science Ethics Committee of the University of Bristol. All participants were volunteers who gave written informed consent.

Experiment 1

### Participants

Fifteen participants (mean age 19.2 years, 11 female, 1 left handed) completed the task. For the 90° condition, only nine participants’ data was analysed. A visual inspection of the data after six participants indicated performance was far better than expected, meaning we may not have had sufficient data points around the JND to estimate it adequately. To address this, we changed the comparison stimuli in the 90° condition from participant seven to increase task difficulty. All participants were undergraduate students from the University of Bristol who were paid £40.

### Materials

A novel set of 3D, triangular prism shapes, with test edges of varying sharpness, were produced using 3D printing technology. The test edge was the edge running along the upper apex of the triangular prism. All shapes shared the same base area of 36×60 mm and had the same height of 26 mm. The sharpness of the test edge was altered by varying the angle at which the two upper surfaces met to form the apex, and this angle, θ, was used as our measure of sharpness.

The shapes were fabricated on an envisionTEC Perfactory 3 printer using RCP30 nano cured ceramic photo polymer. The Perfactory 3 has an XY resolution of 30 µm and a Z resolution of 15 µm. To minimise texture along the test edge, enabling participants to focus on judgements of sharpness alone, shapes were fabricated such that the 60 mm test edge was aligned with the higher resolution Z axis. In subjective handling, the test edges of all shapes were very smooth with no noticeable texture or imperfections.

Eleven shapes were produced with edges that ranged in sharpness from θ = 40° to θ = 90° in 5° increments. We did not produce shapes with edges of sharpness greater than θ = 90°, because exploration of edges with such low sharpness may involve the larger scale sensing of the angle between two separate faces rather than the sharpness of the edge itself. At this time, fabrication is expensive and lengthy, and this further limited the number of shapes we could produce. We selected four shapes distributed approximately evenly across our range of shapes (40°, 50°, 70° and 90°) and used these as references in Experiment 1. For each reference we used seven comparison shapes, giving a range of differences in sharpness, *Δ_θ_,* from 5° to 35° in 5° increments in each condition. Given the relatively limited range of sharpness available, and the lack of a prediction of the likely value of JND, we set the reference to one end of the range of test stimuli in each condition to maximise the chance we would capture the JND. In the θ = 40° and 50° conditions the comparison shapes were all greater in sharpness than the reference, and in the θ = 70° and 90° conditions, they were all less sharp.

### Design and Procedure

**Figure 1:**
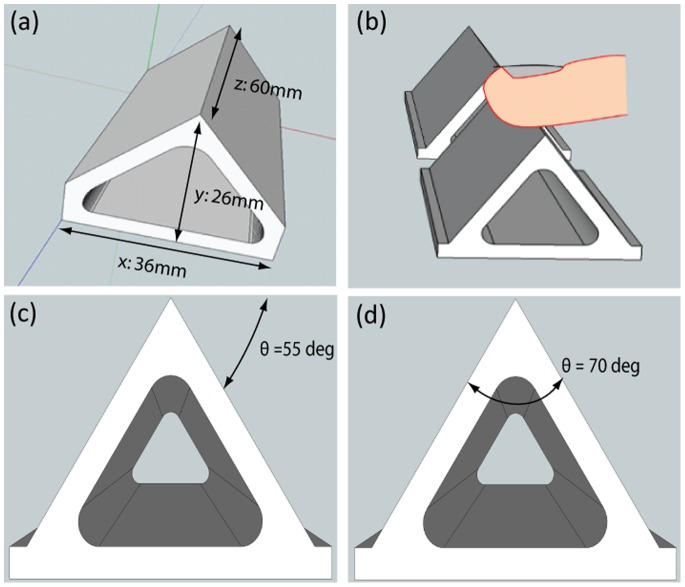
Test stimuli. Shape stimuli (a) illustrated with dimensions and fabrication axes, (b) shown side-by-side as they were presented to the participant, (c) with sharpness θ defined as the angle at which one surface meets the horizontal plane, (d) with sharpness θ defined as the internal angle between the two surfaces.

Participants were blindfolded to prevent perception of visual information from the stimuli, and to ensure non-informative vision had no effect on their haptic perception [31]. On each trial the participant was presented with two shapes with edges that differed in sharpness. One shape was always the reference shape, the other shape was one of the seven comparison shapes. The shapes were located by the experimenter in a flat frame on a table top, using magnets attached to the frame and the bottom of the shapes. The frame ensured the shapes were presented in identical positions across trials, and side by side in front of the participant (see Figure 1). They were asked to explore the left shape first (shape 1), then the right shape (shape 2), and then verbally report which of the two shapes (1 or 2) felt sharper. The beginning of each trial was signalled by an audio tone, and the trials were self-paced. Between trials participants rested their index finger on a raised marker on the table top, located in front of the frame, mid-way between the two shapes.

The method of constant stimuli was used to obtain a set of psychometric data from which the discrimination threshold could be determined. Participants were presented with the reference shape paired with each of the seven comparison shapes 40 times (with position counterbalanced, 20 with the reference shape in the left position, and 20 in the right position), making a total of 280 trials per session. The order of trial presentation was randomised across the session. Each session was divided into four blocks of 70 trials, each lasting approximately 15 minutes, with a rest period between each block. Participants completed the four different reference tasks (one per session) in an order determined by a partial Latin square to minimise order effects.

Participants were instructed to explore the edges of the shapes with a single stationary touch of the fingertip of the index finger of their dominant hand. They were asked to aim their touch at the approximate mid-point of each edge, avoiding the corners of the edge, and to keep the touch static, without any movements that may result in contact with the surfaces of the shape. They were not permitted to return to either shape for a second touch.

### Data Analysis

To estimate the JNDs, we fit a series of psychometric functions to our data. Typically, within the field of haptic perception, the function used has been the logistic. Such a function can model the relationship between proportion correct (*P_correct_*) and sharpness difference (*Δ_θ_*) as
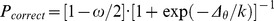
where *ω* is a lapserate parameter that we have included to represent the proportion of responses on which a participant guesses (allowing for asymptotic performance below perfect accuracy).

However, we deliberately sampled our psychometric functions at enough locations, and with enough precision, to build up a good idea of the shape of the relationship between sharpness difference and proportion correct. In addition to the logistic, we therefore used the cumulative Weibull distribution appropriately scaled between 0.5 (chance performance) and 1 (perfect performance) to model our data. Note that scaling the function between 0.5 and 1.0 allows us to shift the curve sideways using its location parameter (*λ*) whilst also being able to adjust the curve’s form using its shape parameter (*k*). This allows us to have a much finer control of the relationship between sharpness difference and proportion correct than that offered by the logistic described above. Therefore, for each participant, in addition to the logistic, we model their data using the following relationship
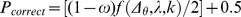
where *f*(*Δ_θ_*, λ, *k*) is the cumulative Weibull distribution at sharpness difference *Δ_θ_*.

We carried out a hierarchical model selection procedure using the Akaike Information Criterion (AIC) to select the model that optimally described our data (balancing goodness-of-fit and the number of free parameters, i.e. model complexity). Models with lower AICs are preferred; the difference between AICs indicates the strength of that preference. We fit each model by maximising their likelihood given the data using Matlab’s *fminsearch* algorithm (based on the Nelder-Mead simplex direct search [32]) to find the minimum deviance. To simplify the search space, we first looked separately at models within the results for each reference set.

Results for both the logistic and Weibull models are shown in Table S1 in the Supporting Information. The model with by far the highest probability is the cumulative Weibull with a separate location parameter for each participant, a shape parameter shared across participants and no lapserate parameter. Note that the logistic function provides a relatively poor description of the data, and we therefore focus on the best fitting Weibull model.

Next, we considered whether parameter values varied across reference set. In this case we have two variations of model, one where the shape parameter is the same across standards and one where it is free to differ across standards. The latter is strongly preferred with an AIC that is 20.9 less than that of the model with shape fixed across standards, suggesting that choice of standard affects the relationship between sensitivity and degree of difference, *Δ_θ_*. The model we therefore use to analyse the discrimination thresholds is a Weibull with no lapsrate, a shape parameter for each reference, and a separate location parameter for each participant. The best fitting model for each individual is shown in Figures S1, S2, S3, and S4 (for references 40°, 50°, 70°, and 90°, respectively) in the Supporting Information.

### Experiment 2

#### Participants

Fourteen participants (mean age 22.6 years, seven female, two left handed) completed the task using the Single Touch strategy, and 15 different participants (mean age 21.5 years, 11 female, one left handed) completed the task using the Free Exploration strategy. All participants were undergraduate students from the University of Bristol who participated for course credit.

#### Materials

The 70° reference set of stimuli from Experiment 1 were used (stimuli differed from 5° to 35° in steps down of 5° from the 70° referent).

#### Design and procedure

The design and procedure were identical to Experiment 1, except participants were only exposed to the 70° reference set and they either explored with a Single Touch strategy or a Free Exploration strategy. The single Touch strategy was identical to the procedure used to explore the edges in Experiment 1.

The procedure for the Free Exploration strategy was identical to that used in the Single Touch strategy, with the only exception that participants were free to use a different strategy to explore the edges of the shapes. They were again instructed to explore the edges of the shapes with the fingertip of the index finger of their dominant hand, and to avoid touching the corners of the edge. But with the Free Exploration strategy they were not constrained in terms of the way they should touch the shapes, the number of touches they should use, or the order in which to explore the shapes. They were allowed to move their exploration between the two shapes at will, as long as they continued to avoid the corners of the edges, in particular those at the boundary of the two shapes.

#### Data analysis

We completed an analysis similar to that of Experiment 1, fitting both the logistic and Weibull functions to the individual participants’ data. To simplify the search space, we first looked separately at models within the results for each exploration strategy. Results for both the logistic and Weibull models are shown in Table S2 in the Supporting Information.

The best fitting model was the same as Experiment 1: A Weibull with no lapsrate, a separate location parameter for each participant and a shape parameter shared across participants. This model fit six times as well as the next best fitting model (which included the lapserate). Whilst the latter is still a reasonable candidate, we note that the estimated lapserates are small (0.0012 for the Single Touch strategy and 0.0085 for the Free Exploration strategy) and make little difference to the other parameters. Next, we considered whether parameter values varied between exploration strategies. In this case we have two variations of the model, one where the shape parameter is the same across strategies and one where it is free to differ across strategies. The latter is strongly preferred with an AIC that is 18.2 less than that of the model with shape fixed across strategies, suggesting that exploration strategy affects the relationship between sensitivity and degree of difference, *Δ_θ_*. We use this model to analyse the thresholds and the best fitting model for each individual participant is shown in Figure S5 and S6 for the Single Touch and Free Exploration Strategies, respectively.

As a useful guide for future experiments using sharpness that need an estimate of a shape parameter (e.g., trying to estimate thresholds efficiently, as in the QUEST method), we fitted the Weibull across all our data sets from Experiment 1 and Experiment 2 with the shape parameter fixed to provide an estimate of the best-guess shape parameter to use. The resulting shape value was 1.07 (with a 95% confidence interval of 1.00–1.16, calculated with 10,000 bootstrapped iterations using the percentile method [33].

## Results

### Experiment 1

**Figure 2:**
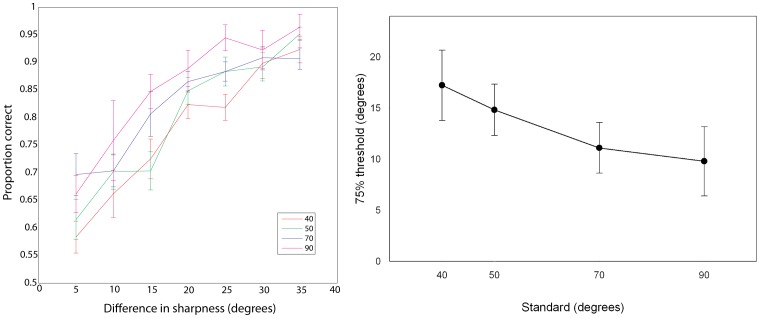
Results from Experiment 1. Left Panel: Proportion correct averaged across individuals for each degree difference between referent and standard, for the four reference sets. Right panel: Mean 75% thresholds calculated using the best fitting Weibull function for each referent in Experiment 1. Error bars are 95% confidence intervals.

The averaged data for each reference set and degree difference *Δ_θ_*, is shown in Figure 2 (left panel) which shows the typical increase in accuracy as difference between the standard and reference increases for all four reference sets. We fitted our model to the data from individual participants to compare discrimination thresholds across our four groups of participants. The data for individual participants are provided in file Dataset S1 and in Figures S1, S2, S3, and S4 in the Supporting Information. The mean 75% thresholds for the four references are shown in Figure 2 (right panel), which shows that there is a reduction in threshold as the standard is increased, with mean thresholds of 17.2°, 14.8°, 11.1° and 9.8° for references θ = 40°, 50°, 70° and 90° respectively.

As discussed, to compare our JNDs across studies and different physical measures we can express these as Weber fractions, which are 0.43, 0.30, 0.15 and 0.11 for the θ = 40°, 50°, 70° and 90° conditions, respectively. Wijntjes and Kappers [15] study of 2D angle discrimination reported Weber fractions of 0.3 for a reference of 20° decreasing to 0.08 for a reference of 135°. Our thresholds follow a broadly similar, non-Weberian pattern decreasing from 0.43 for a reference of 40° to 0.11 for a reference of 90°. However, our measure of sharpness does not vary in the horizontal plane, but in the vertical plane. As such, a more appropriate comparison may be to the 2D angle discrimination thresholds reported by Voisin and colleagues [14]. They reported Weber fractions of around 0.05 for a reference of 90°. This suggests a considerably greater sensitivity to 2D angle discrimination in those studies than to changes in sharpness in the current study.

However, caution is again required in making such comparisons. Participants in the Voisin studies [14], [22], [24] explored the faces of a concave stimulus to make comparisons between 2D angles. Furthermore, because the concave stimulus was V shaped, participants would have been unlikely to sample the apex where the two angled sides meet in their exploration. In the current study, the opposite was true: participants explored the vertex of the edge but not the surfaces forming the edge. The most informative comparison here may not be to studies of 2D angle discrimination, but to studies exploring discrimination of curvature. In both cases, the aspect of the stimulus that is of interest (the curvature and the sharpness) can be sensed directly by cutaneous mechanisms alone (although proprioceptive mechanisms do play a role in curvature discrimination [22]).

The measure of sharpness we adopted in order to make the initial comparisons with studies of 2D angle discrimination makes comparing results from studies of curvature difficult. The current measure, the internal angle between the two sides forming the edge, gives a value that *decreases* as the edge become sharper. Curvature is calculated as the reciprocal of the radius of the curve, and therefore gives a measure that *increases* as the surface becomes more curved. To make a meaningful comparison, we would need a measure of sharpness that, like the measure of curvature, increases monotonically as the tactile psychological percept of sharpness increases.

An alternative to our original approach of measuring sharpness as the internal angle between the two sides of the edge is to redefine sharpness as the open angle formed between one surface and the horizontal plane, as shown in Figure 1. This gives a value of sharpness that monotonically increases with angle θ. Adopting this new measure requires no changes to the model fitting part of the analysis: the relationships between sensitivity and change in sharpness across standards remain unchanged. It does mean, however, that the same change in sharpness of our stimuli in sharpness now has a value *Δ_θn_* half the value it did originally. The values of our references become 45°, 55°, 65°, and 70°, for 90°, 70°, 50°, and 40°, respectively. The corresponding thresholds in these conditions are now 4.9°, 5.6°, 7.4° and 8.6° and corresponding Weber fractions of 0.11, 0.10, 0.11 and 0.12. Using this measure of sharpness, we can see our results come close to following Weber’s Law, in which the fraction of threshold and reference (i.e. the Weber fraction) is constant across different levels of reference. Following the approach of Park and colleagues [7], a single Weber fraction for all four reference conditions can be derived by plotting mean discrimination thresholds as a function of reference sharpness. The slope of the best fitting line that passes through the origin is the overall Weber fraction. With our revised measure of sharpness our Weber fraction for Experiment 1 was 0.11.

Park et al. [7], in their review of discrimination of curvature using real (rather than virtual) stimuli indicated that the Weber fractions for these were in the range 0.08 to 0.11. We can see that, when we adopt a measure of sharpness that (as with the measure of curvature) increases as the psychological percept of the material property of interest increases, our overall Weber fraction for discrimination of edge sharpness of 0.11 sits at one end of this range. This suggests that the sensitivity of our haptic system to changes in sharpness is, in broad terms, equivalent to its sensitivity to changes in curvature.

### Experiment 2

**Figure 3:**
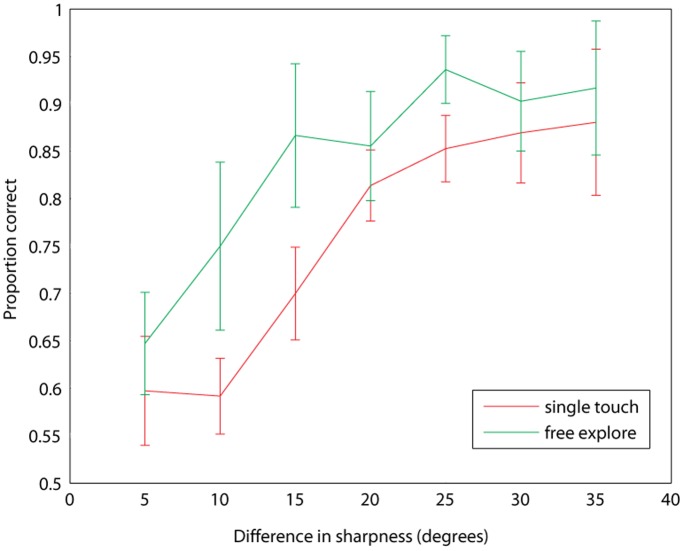
Results from Experiment 2. Proportion correct averaged across individuals for each degree difference between referent and standard, for the Single Touch (red line) and Free Exploration (green line) strategies. Error bars are 95% confidence intervals.

Figure 3 shows the averaged accuracy for the Single Touch and Free Exploration strategies as a function of degree difference *Δ_θ._* The typical increase in accuracy with increased *Δ_θ_* is evident. The data for individual participants are provided in file Dataset S2 and shown in Figures S5 and S6 in the Supporting Information. We fitted the preferred model to our data to look for differences in thresholds between our two groups. As in Experiment 1, the difference in sharpness that corresponded to a performance level of 75% was taken as the discrimination threshold for each participant. For the Single Touch strategy, the mean threshold was 8.6°, and for the Free Exploration strategy, the mean was significantly lower at 5.0° (*t*(27) = 2.63, *p* = .014).

The decreased threshold using the Free Exploration strategy is expected, because an unconstrained exploration provides many opportunities to gather more information than a single touch, enabling more accurate discrimination and a consequent lowering of discrimination threshold. A similar dependence of discrimination threshold on exploration strategy has been observed in many previous studies of haptic discrimination [30]. Nonetheless, it is important to establish the magnitude of difference between unconstrained and minimal exploration techniques in order to gain a better understanding not only of the limits of haptic perception of sharpness, but also to understand better how techniques will affect the information derived from interaction with haptic displays. Anecdotal reports from participants using the Free Exploration strategy suggested three types of exploration were particularly informative.

The first was simply to touch the edges more than once. A multiple touch advantage has been reported in many investigations of haptic performance, with, for example, a reduction in texture discrimination thresholds [34], and an improved recognition of shape contours [35] with increases in the number of touches.

The second type of exploration was to move the glabrous skin of the fingertip in a slow, fore and aft movement over the edge. Interestingly, this is similar to the particular exploration procedure Lederman and Klatzky [36] suggested is optimal in the haptic exploration of surface texture. In this, the movement of fingers tangentially across a surface activates both Merkel neurite SA 1 [37] and Pacinian Corpuscle (PC; [38]) mechanoreceptors. These two cutaneous neural systems are believed to be responsible for perception of roughness, with Merkel SA 1 receptors responding to macro-scale textural stimulation, and PC receptors to micro-scale textural stimulation [30], [38], [39]. In comparison, the cutaneous information contributing to discrimination of curvature (using just the fingertip) comes primarily from Merkel SA 1 mechanoreceptors [40]. Discrimination of the sharpness of real ‘sharp’ edges may therefore differ from discrimination of curvature in the use of additional, fine grained information about texture from PC afferents. In many ways this makes sense if we consider sharpness as the texture of an edge. Future studies will need to examine in more detail the relationship between texture perception and sharpness perception.

The third type of exploration strategy was to slide the glabrous skin of the fingertip along the edge of the shape in a lateral movement. This may produce a similar sensation as that from the fore-aft movement. We are conducting further investigations of these three possible exploration strategies to determine which of them (or which combination) leads to the improved discriminability.

## Discussion

The current studies demonstrated that thresholds varied as a function of the reference, with lower thresholds for larger angles. Using a free exploration strategy as opposed to a ‘tactile glance’ significantly lowered the threshold. The Weber fraction for sharpness (calculated using the angle at which one face meets the horizontal plane) was 0.11.

Our data analysis clearly identified the Weibull as the most appropriate model to use. However, there is inconsistency in the haptic literature both about which model to use (with some researchers preferring the logistic or the normal, without testing which function provides a better description) and about which threshold to use (we used the 75% threshold here in order to draw comparisons with previous studies, but for the Weibull a useful value is 81.6% as no matter the value of the shape parameter, all curves with the same location parameter will pass through this point). Thus, it is often difficult to compare results across studies. Compounding the problem is the fact that the Weber fraction (which allows cross-dimension comparisons) is dependent on the measure which is chosen to represent the magnitude of a stimulus. In our study, using the definition of sharpness as the internal angle formed by the junction of two faces results in a larger Weber fraction (suggesting relatively poor performance) than using our second measure of sharpness, the angle formed by one face and the horizontal plane. The latter preferred measure leads to an increase in sharpness with an increasing angle and produces a relatively constant Weber fraction over the range of thresholds. Using this measure essentially divides our thresholds in half, leading to ‘better’ performance. Both measures of sharpness are legitimate, but lead to different conclusions when considering the relative discriminability of sharpness compared with, for example, 2D angle as measured by Voisin and colleagues [14], [22], [24]. Thus, one must be careful when making cross-study and cross-dimension comparisons. Notwithstanding these concerns, it is helpful to put performance in our study in the context of other haptic discriminability studies in order to decide whether or not our findings tell us much about the utility of edge sharpness in haptic devices. The findings suggest our sensitivity to changes in edge sharpness is lower than but not qualitatively different to our sensitivity to changes in curvature, but less sensitive than 2D angle perception. The Weber fraction observed in Experiment 1 (0.11) is less than those for amplitude of vibration (0.25 [41]), which is a means of conveying information used routinely in haptic interfaces. Thus, edge sharpness may well prove useful for providing and signalling information in the haptic modality. Recent advances in material science mean self-actuated materials that can adapt their shapes in a variety of ways will soon be available in haptic interfaces. These materials can alter the curvature of shapes they produce [42] and at some point may be able to produce interactive haptic elements that vary in sharpness too [43].

In the real-world, two surfaces will rarely meet to form a perfect, sharp edge, and the edge formed will be curved to some degree. This means the sharpness of real edges may best be considered as having two components, namely, the angle at which the surfaces meet and the curvature at the point the surfaces meet. Teasing apart these two components and investigating our sensitivity to these independently is likely to be a worthwhile endeavour for future investigations.

Sharpness thresholds, like other tactile thresholds, will be affected, not only by the characteristics of the edges themselves, but also by the characteristics of the skin touching the edge. The distribution of mechanoreceptors in the skin covering different parts of the body varies considerably and these differences will result in different thresholds in haptic spatial discrimination [44] and temporal discrimination [45]. This means we can expect the discrimination thresholds for sharpness to vary from one part of the body to another. The current study, along with others described here, measured thresholds on the glabrous skin of the index finger: the same thresholds measured on the skin of the arm or the back may well be very different. This will clearly be an important factor in the design of any haptic interface that modulates edge sharpness.

In summary, many devices with haptic interfaces remain unused, often because they continue to be designed without knowledge of the limitations of our haptic system [46]. The results from this study of the discrimination of haptic edge sharpness, and those in which we are currently engaged, will aid the design and engineering of future haptic devices. Further, it is clear that edges play a key role in haptic perception: edges are, after all, primitives of shape. Understanding how our haptic system responds to the different characteristics of edges is therefore an important part of developing a complete picture of our haptic perception system.

## Supporting Information

Figure S1 **Data from Experiment 1 using a 40 degree standard, showing proportion of correct responses as difference in sharpness between reference and test shape varies.** Curve shown is best fitting cumulative Weibull function.
(TIF)

Figure S2 **Data from Experiment 1 using a 50 degree standard, showing proportion of correct responses as difference in sharpness between reference and test shape varies.** Curve shown is best fitting cumulative Weibull function.
(TIF)

Figure S3 **Data from Experiment 1 using a 70 degree standard, showing proportion of correct responses as difference in sharpness between reference and test shape varies.** Curve shown is best fitting cumulative Weibull function.
(TIF)

Figure S4 **Data from Experiment 1 using a 90 degree standard, showing proportion of correct responses as difference in sharpness between reference and test shape varies.** Curve shown is best fitting cumulative Weibull function.
(TIF)

Figure S5 **Data from Experiment 2 using a 70 degree standard and single touch exploration strategy, showing proportion of correct responses as difference in sharpness between reference and test shape varies.** Curve shown is best fitting cumulative Weibull function.
(TIF)

Figure S6 **Data from Experiment 2 using a 70 degree standard and free exploration strategy, showing proportion of correct responses as difference in sharpness between reference and test shape varies.** Curve shown is best fitting cumulative Weibull function.
(TIF)

Table S1 **Model selection for Experiment 1 using AICs.**
(DOCX)

Table S2 **Model selection for Experiment 2 using AICs.**
(DOCX)

Dataset S1 **Tab-delimited text file, with column headings, containing the complete data from Experiment 1.**
(TXT)

Dataset S2 **Tab-delimited text file, with column headings, containing the complete data from Experiment 2.**
(TXT)
