# Supplementary figures and images for: On the Edge: Haptic Discrimination of Edge Sharpness
Source: PLoS One. 2013 Sep 4;8(9):e73283. doi: 10.1371/journal.pone.0073283 (PMC3762717; doi:10.1371/journal.pone.0073283)

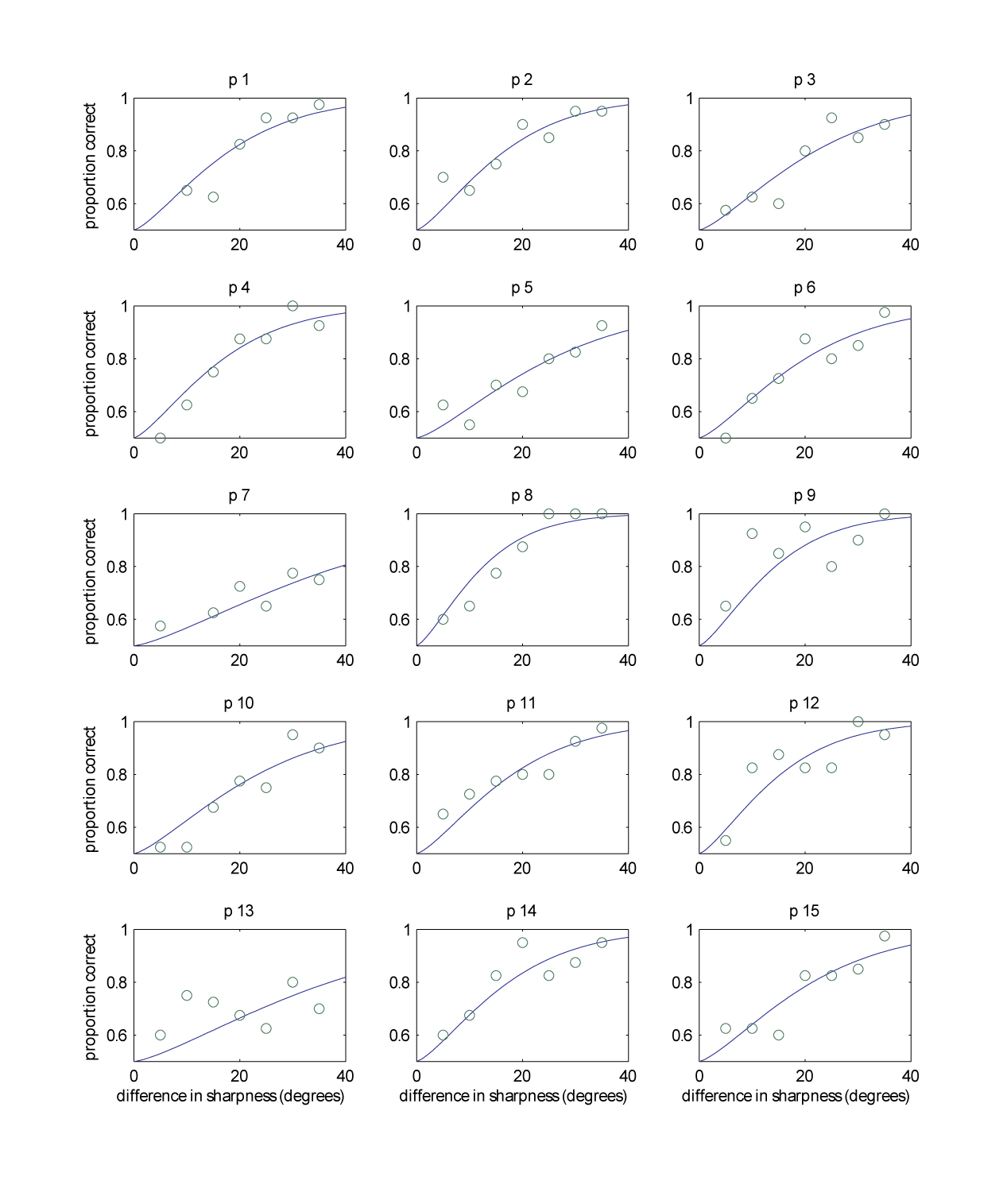

Supplement: Figure S1 — Data from Experiment 1 using a 40 degree standard, showing proportion of correct responses as difference in sharpness between reference and test shape varies. Curve shown is best fitting cumulative Weibull function. (TIF) [file pone.0073283.s001.tif]

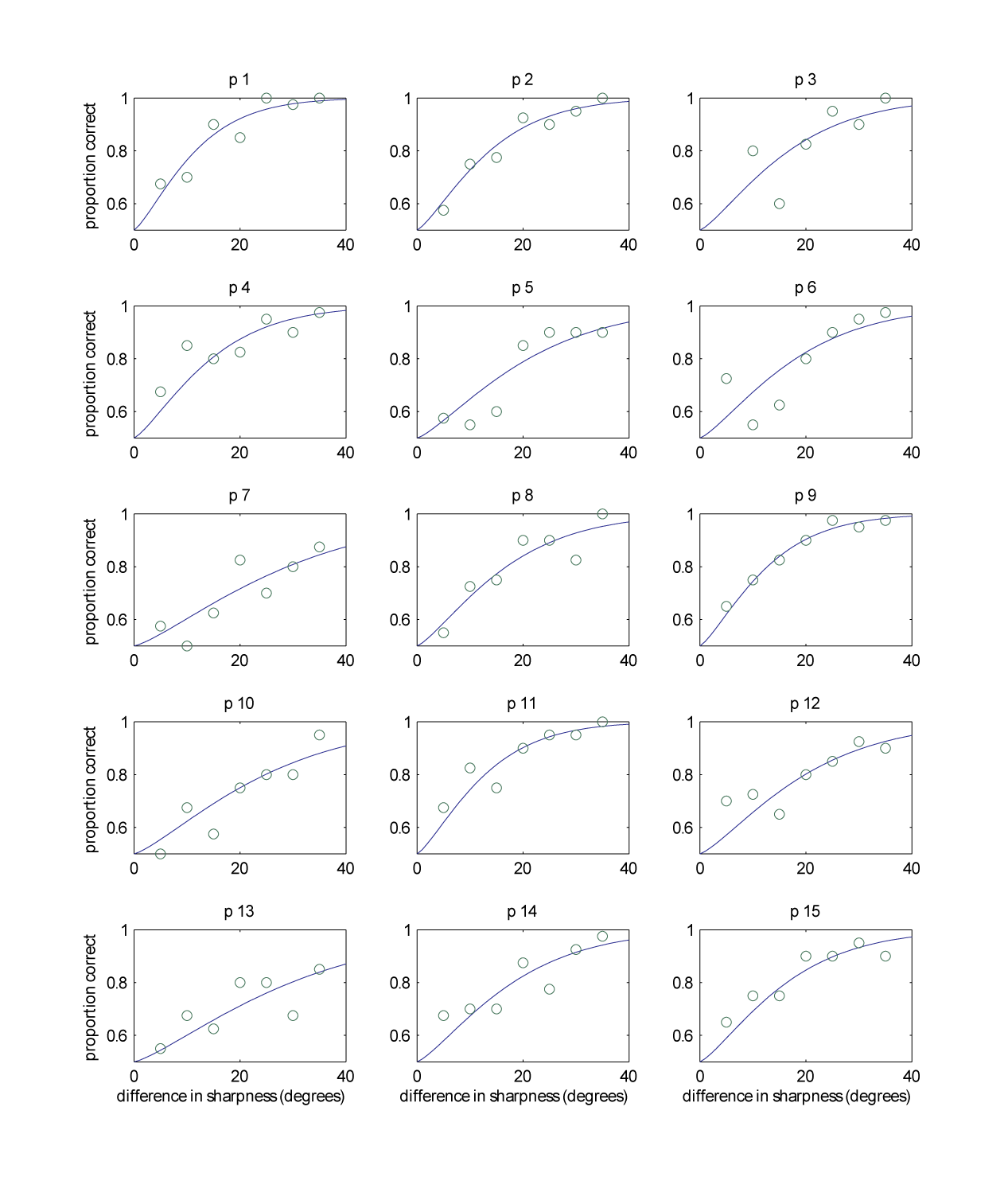

Supplement: Figure S2 — Data from Experiment 1 using a 50 degree standard, showing proportion of correct responses as difference in sharpness between reference and test shape varies. Curve shown is best fitting cumulative Weibull function. (TIF) [file pone.0073283.s002.tif]

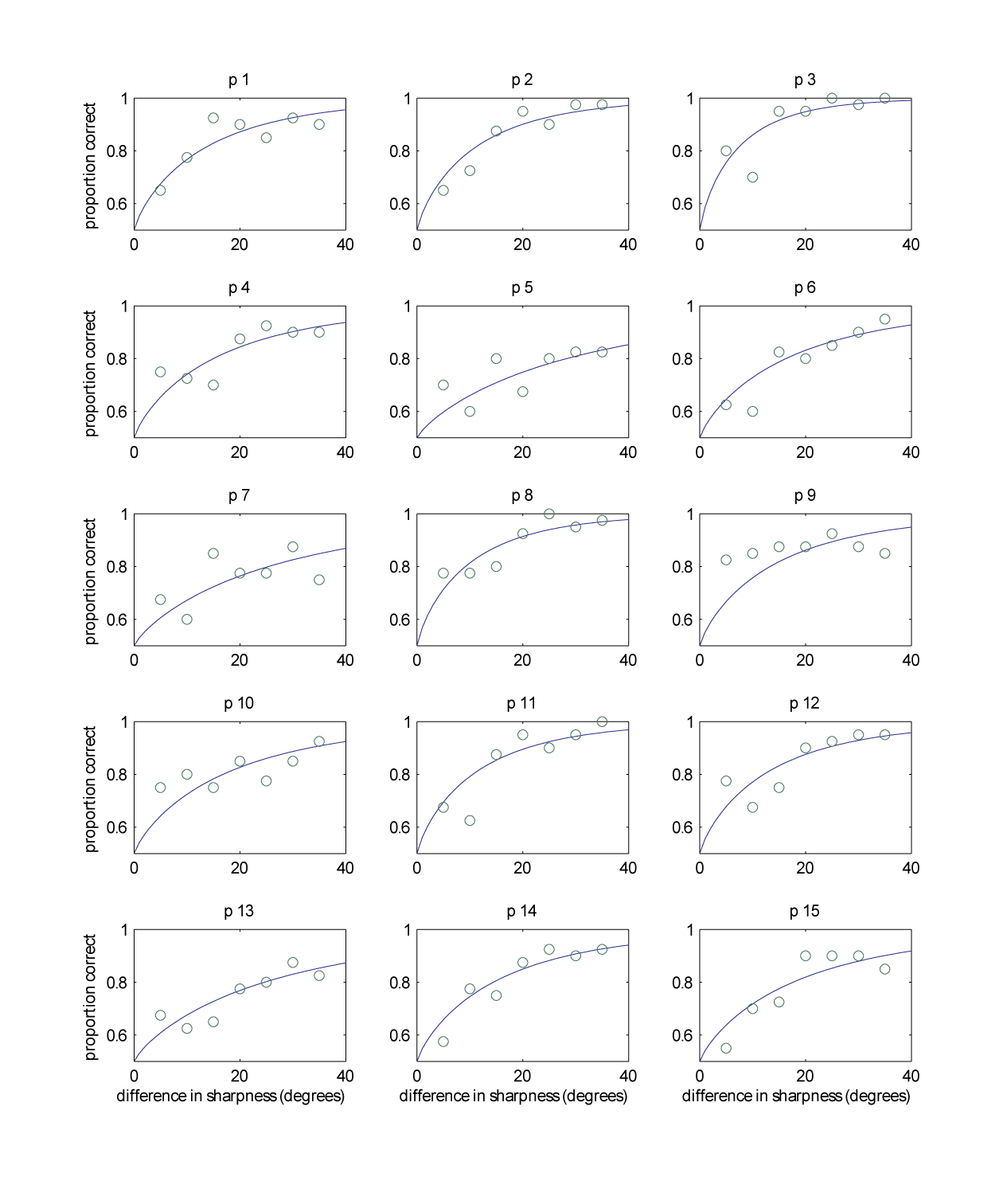

Supplement: Figure S3 — Data from Experiment 1 using a 70 degree standard, showing proportion of correct responses as difference in sharpness between reference and test shape varies. Curve shown is best fitting cumulative Weibull function. (TIF) [file pone.0073283.s003.tif]

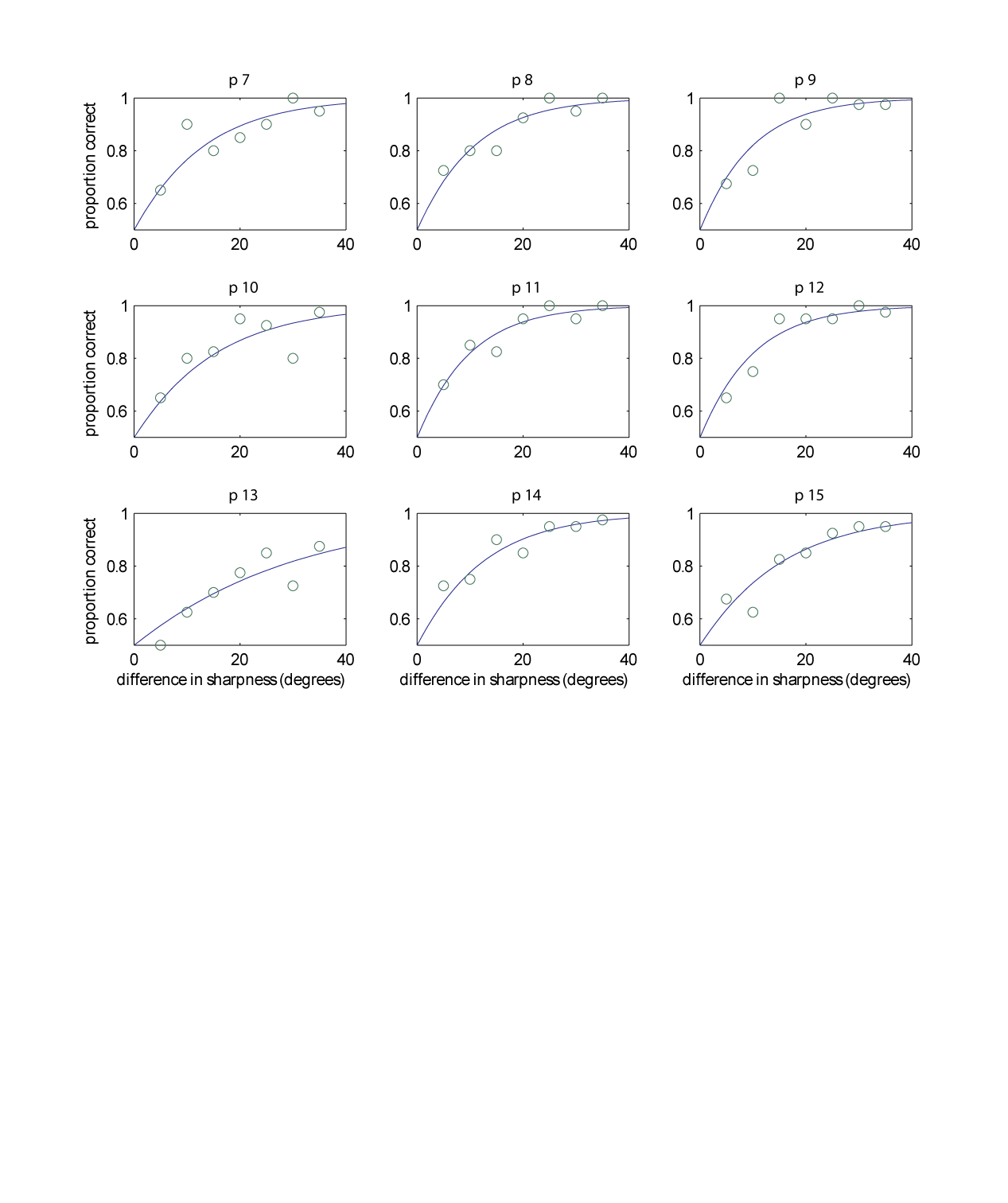

Supplement: Figure S4 — Data from Experiment 1 using a 90 degree standard, showing proportion of correct responses as difference in sharpness between reference and test shape varies. Curve shown is best fitting cumulative Weibull function. (TIF) [file pone.0073283.s004.tif]

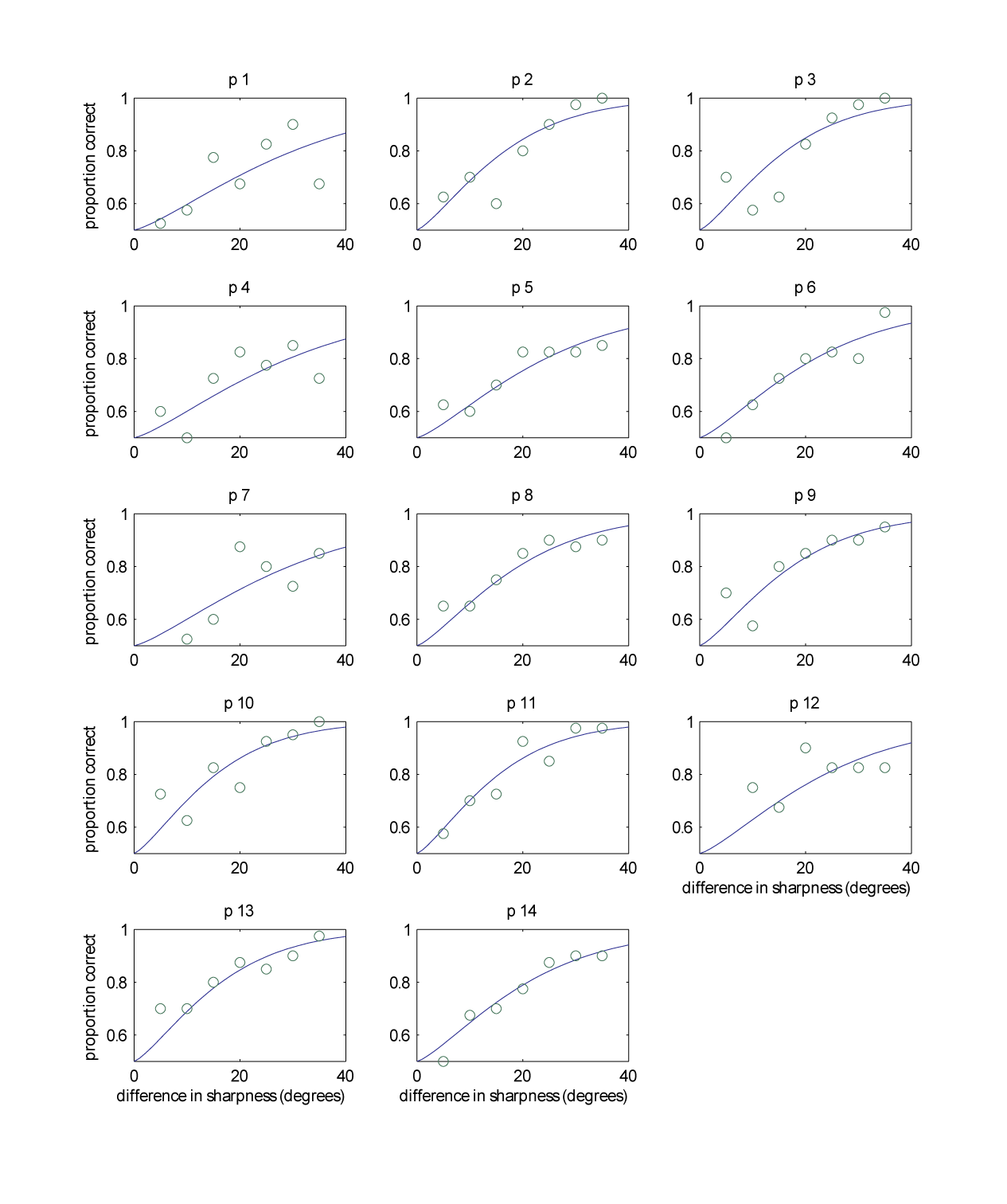

Supplement: Figure S5 — Data from Experiment 2 using a 70 degree standard and single touch exploration strategy, showing proportion of correct responses as difference in sharpness between reference and test shape varies. Curve shown is best fitting cumulative Weibull function. (TIF) [file pone.0073283.s005.tif]

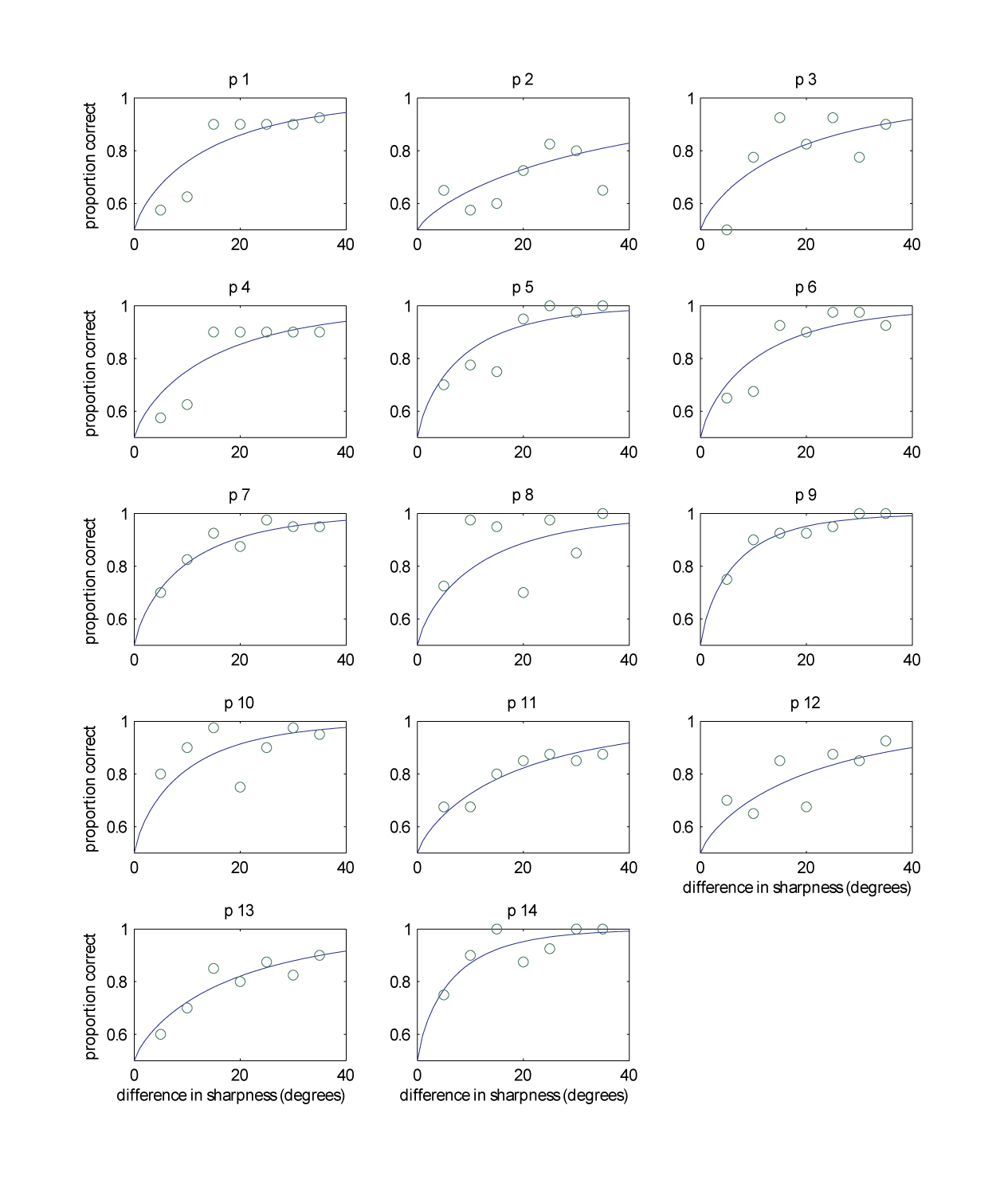

Supplement: Figure S6 — Data from Experiment 2 using a 70 degree standard and free exploration strategy, showing proportion of correct responses as difference in sharpness between reference and test shape varies. Curve shown is best fitting cumulative Weibull function. (TIF) [file pone.0073283.s006.tif]
