# Supplementary material for: On the Edge: Haptic Discrimination of Edge Sharpness
Source: PLoS One. 2013 Sep 4;8(9):e73283. doi: 10.1371/journal.pone.0073283 (PMC3762717; doi:10.1371/journal.pone.0073283)
Supplement: Table S1 — Model selection for Experiment 1 using AICs. (DOCX) [file pone.0073283.s007.docx]

Table S1. Model selection for Experiment 1 using AICs.

| Location | Shape | Lapserate | ΔAIC_40_ | ΔAIC_50_ | ΔAIC_70_ | ΔAIC_90_ | ΔAIC_Σ_ | *w*(ΔAIC_Σ_) |
| --- | --- | --- | --- | --- | --- | --- | --- | --- |
|  | 0 | 0 | 84.4 | 72.4 | 73.0 | 49.9 | 280 | .000 |
|  | 1 | 0 | 4.49 | 31.2 | 29.7 | 17.6 | 83.1 | .000 |
|  | 0 | 1 | 111 | 100 | 105 | 73.4 | 391 | .000 |
|  | 1 | 1 | 30.4 | 85.8 | 57.5 | 17.9 | 192 | .000 |
|  | 0 |  | 82.4 | 70.4 | 96.4 | 55.8 | 305 | .000 |
|  | 1 |  | 13.6 | 16.6 | 141 | 20.1 | 191 | .000 |
| 0 | 0 | 0 | 86.4 | 75.4 | 64.9 | 50.9 | 278 | .000 |
| 1 | 0 | 0 | 2.13 | 2.11 | 23.1 | 2.01 | 29.4 | .000 |
| 0 | 1 | 0 | 68.9 | 75.1 | 35.3 | 31.8 | 211 | .000 |
| 0 | 0 | 1 | 114 | 56.5 | 49.5 | 12.4 | 233 | .000 |
| 1 | 1 | 0 | 5.30 | 22.2 | 5.17 | 16.9 | 49.6 | .000 |
| 1 | 0 | 1 | 30.0 | 30.0 | 29.6 | 18.0 | 108 | .000 |
| 0 | 1 | 1 | 115 | 109 | 63.5 | 48.1 | 335 | .000 |
| 1 | 1 | 1 | 35.1 | 51.0 | 43.5 | 30.2 | 160 | .000 |
| 0 | 0 |  | 84.4 | 73.4 | 63.0 | 49.4 | 270 | .000 |
| 1 | 0 |  | 0 | 0 | 0 | 0 | 0 | 1.00 |
| 0 | 1 |  | 67.5 | 73.2 | 34.0 | 30.1 | 205 | .000 |
| 1 | 1 |  | 3.95 | 13.1 | 8.40 | 18.2 | 43.6 | .000 |

*Note.* The first three columns describe the models used with a 0 indicating a parameter shared across participants and a 1 indicating a parameter free to vary across participants. No value indicates that this parameter was not employed in the model. The topmost six rows, with blank location parameters, show results for the logistic fits. The remaining rows show results for the cumulative Weibull. The 4th to 7th columns shows AIC differences for the various standards, the 8th column shows AIC differences for the AICs summed across standards. The final column shows the Akaike weights derived from that penultimate column.
