# Supplementary material for: On the Edge: Haptic Discrimination of Edge Sharpness
Source: PLoS One. 2013 Sep 4;8(9):e73283. doi: 10.1371/journal.pone.0073283 (PMC3762717; doi:10.1371/journal.pone.0073283)
Supplement: Table S2 — Model selection for Experiment 2 using AICs. (DOCX) [file pone.0073283.s008.docx]

Table S2. Model selection for Experiment 2 using AICs.

| Location | Shape | Lapserate | ΔAIC_Single_ | ΔAIC_Free_ | ΔAIC_Combined_ | *w*(ΔAIC_Combined_) |
| --- | --- | --- | --- | --- | --- | --- |
|  | 0 | 0 | 36.7 | 115 | 152 | .000 |
|  | 1 | 0 | 1.11 | 55.5 | 56.6 | .000 |
|  | 0 | 1 | 46.1 | 188 | 234 | .000 |
|  | 1 | 1 | 27.0 | 58.4 | 85.4 | .000 |
|  | 0 |  | 35.9 | 159 | 195 | .000 |
|  | 1 |  | 2.73 | 107 | 110 | .000 |
| 0 | 0 | 0 | 36.9 | 112 | 149 | .000 |
| 1 | 0 | 0 | 2.08 | 1.58 | 3.67 | .138 |
| 0 | 1 | 0 | 31.5 | 74.2 | 106 | .000 |
| 0 | 0 | 1 | 65.4 | 94.0 | 159 | .000 |
| 1 | 1 | 0 | 9.66 | 12.6 | 22.3 | .000 |
| 1 | 0 | 1 | 30.3 | 28.1 | 58.4 | .000 |
| 0 | 0 |  | 37.5 | 112 | 149 | .000 |
| 1 | 0 |  | 0 | 0 | 0 | .862 |
| 0 | 1 |  | 30.4 | 84.3 | 115 | .000 |
| 1 | 1 |  | 14.6 | 11.7 | 26.3 | .000 |

*Note.* The first three columns describe the models used with a 0 indicating a parameter shared across participants and a 1 indicating a parameter free to vary across participants. No value indicates that this parameter was not employed in the model. The fourth column shows AIC differences for the Single Touch strategy, the fifth column shows AIC differences for the Free Exploration strategy. The sixth column shows these combined whilst the final column shows the Akaike weights derived from that penultimate column.
